# CCTA of Pediatric Congenital Right Heart Obstructive Lesions: A Pictorial Review

**DOI:** 10.3390/diagnostics16131959

**Published:** 2026-06-24

**Authors:** Zuofeng Zheng, Lei Xu

**Affiliations:** Department of Radiology, Beijing Anzhen Hospital, Capital Medical University, Beijing 100029, China

**Keywords:** congenital heart disease, right heart obstruction, CCTA, inflow obstruction, outflow obstruction

## Abstract

Pediatric congenital right heart obstructive lesions encompass a spectrum of diseases that obstruct blood flow from the right atrium to the pulmonary artery. Right ventricular inflow obstructions include tricuspid valve abnormalities, such as Ebstein anomaly, tricuspid valve dysplasia, and tricuspid atresia. Right ventricular outflow obstructions include pulmonary valve stenosis, pulmonary atresia, and tetralogy of Fallot. Cardiac computed tomography angiography (CCTA) is a valuable tool for the diagnosis, treatment planning, and follow-up of these lesions. In this pictorial review, we highlight the diagnostic utility of CCTA in congenital right heart obstructive lesions, emphasizing its role in preoperative planning.

## 1. Introduction

In pediatric congenital heart disease (CHD), right heart obstructive lesions represent a heterogeneous group of structural anomalies that obstruct blood flow at any point from the right atrium to the pulmonary artery. These lesions can be broadly classified into inflow obstructions, such as tricuspid valve abnormalities, and outflow obstructions, such as various stenoses of the right ventricular outflow tract (RVOT). They can occur in isolation or in association with other congenital anomalies. The clinical presentation varies widely, ranging from asymptomatic cases to cyanosis, arrhythmias, and even severe right heart failure, depending on the degree of obstruction and associated anomalies [1].

Echocardiography remains the first-line imaging modality for pediatric CHD, because of its real-time hemodynamic assessment, absence of ionizing radiation, and wide availability. However, it has significant limitations: operator dependence, limited acoustic windows, poor visualization of extracardiac vessels (e.g., distal pulmonary arteries, aortopulmonary collaterals, or coronary anomalies), and difficulty in assessing lung parenchyma or airway compression. Cardiac magnetic resonance imaging (MRI) offers excellent soft-tissue contrast and flow quantification without ionizing radiation. However, it requires long acquisition times, often needs sedation in young children, and has lower spatial resolution for small-vessel anomalies. As the gold standard for angiography, digital subtraction angiography (DSA) is invasive and carries radiation and contrast-related risks, so it is not recommended for routine screening or regular follow-up.

Cardiac computed tomography angiography (CCTA) has emerged as a critical imaging modality for the evaluation of congenital heart disease (CHD). Unlike echocardiography, CCTA provides high-resolution, three-dimensional anatomical delineation of complex cardiac and vascular structures, including the RVOT, pulmonary valve, and branch pulmonary arteries, which are often challenging to assess with other imaging techniques [2]. Additionally, CCTA offers superior spatial resolution for detecting associated anomalies, such as coronary artery anomalies or aortopulmonary collaterals. These advantages make it an effective complementary modality to echocardiography and MRI in complex anatomical assessment and preoperative planning [1]. The purpose of this pictorial review is to review the spectrum of abnormalities of congenital right heart obstructive lesions detected by means of CCTA in pediatric patients.

## 2. Pathophysiology of Congenital Right Heart Obstructive Lesions

Congenital right heart obstructive lesions are characterized by impaired blood flow through the right heart to the pulmonary circulation. These alterations prompt compensatory responses to sustain pulmonary artery perfusion. To bypass this “dead end,” deoxygenated systemic venous blood may be forced through any existing communication—such as an atrial septal defect (ASD), ventricular septal defect (VSD), or a patent foramen ovale (PFO)—into the left heart. This right-to-left shunt results in the mixing of deoxygenated blood with oxygenated blood in the systemic circulation, causing central cyanosis [3]. A left-to-right shunt can also occur, primarily in cases of tricuspid atresia (TA), where blood flows from the right atrium through an ASD into the left heart and then passes via a VSD to the right ventricle and pulmonary artery. When a VSD is not present or there is significant pulmonary obstruction, pulmonary blood flow (PBF) is typically maintained via a patent ductus arteriosus (PDA) or aortopulmonary collateral vessels, ensuring adequate oxygenation. In these patients, the severity of cyanosis is determined by the degree of PBF, which depends on several factors: the extent of pulmonary obstruction, the presence and size of a VSD, the relationship between the great arteries, and whether a PDA is present [4].

## 3. Technical Considerations of CCTA in Pediatric Congenital Right Heart Obstructive Lesions

Given children’s small body size, fast heart rate, limited breath-holding ability and variable cardiac anatomy, one-size-fits-all protocols are not applicable. All scanning parameters must be individualized to balance diagnostic image quality and minimal radiation exposure. Sedation or anesthesia should also be avoided whenever possible. Cardiac gating, the main technique for reducing motion artifacts, is essential for pediatric cardiac CCTA when assessing coronary artery anomalies and visualizing full coronary vessels. Beta-blockers are rarely used in pediatric patients. Prospective ECG gating is preferred for lower radiation, while retrospective gating is reserved for detailed coronary or ventricular assessment. Modern dual-source and 320-row volumetric CT scanners effectively reduce motion artifacts and often eliminate the need for breath-holding or sedation. Iodinated contrast is administered at 1.5–2 mL/kg (max 3 mL/kg) with tailored injection rates and scan timing based on cardiac anatomy and pathology. Radiation dose is optimized by adjusting kV/mA, limiting scan range and adopting iterative reconstruction [5]. However, compared with cardiac catheterization, CCTA delivers considerably less radiation and carries a low risk of contrast medium-related adverse reactions. Strategies like feed-and-wrap scanning can also be utilized to eliminate the requirement for sedation [6]. Special protocols including delayed equilibrium-phase scanning are needed for patients with Fontan circulation [5].

Recent studies demonstrate that photon-counting CT (PCCT) has unique advantages in imaging pediatric CHD. PCCT achieves lower radiation dose than 256-slice (Energy-Integrating Detector CT) EID-CT for CHD imaging, and provides higher signal-to-noise ratio (SNR)/contrast-to-noise ratio (CNR) and better pediatric cardiac image quality than dual-source CT at identical radiation doses [6,7].

## 4. Right Ventricular Inflow Obstructions

### 4.1. Ebstein Anomaly (EA)

**Figure 1:**
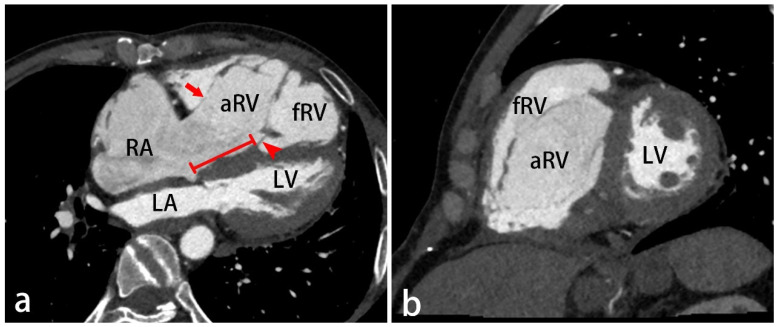
A 17-year-old male with Ebstein anomaly. Four-chamber view (**a**) and two-chamber view (**b**) of CT angiography shows inferior displacement of the septal leaflets of the tricuspid valve ((**a**), red arrowhead), resulting in an atrialized right ventricle and reduction in the size of the functional right ventricle. The degree of functional tricuspid valve annular displacement can be quantified by measuring the distance between the hinge points of the septal leaflet and the anterior leaflet of the mitral valve ((**a**), red line). The antero-superior leaflet has multiple attachments to the right ventricle wall, showing “sail-like” appearance ((**a**), red arrow). LV, left ventricle; LA, left atrium; RA, right atrium; aRV, atrialized right ventricle; fRV, functional right ventricle.

EA accounts for approximately 0.5% of all CHDs [8]. Anatomically, the hallmark of EA is the inferior displacement of the septal and posterior leaflets of the tricuspid valve (TV) away from the atrio-ventricular junction and into the right ventricle [9]. The characteristic embryological defect in EA is the failed delamination of the TV leaflets from the underlying primitive right ventricular myocardium. This results in abnormal tethering of the TV leaflets to the right ventricular free wall and septum, which in turn causes the characteristic downward displacement of the annular attachments (hinge points) of the septal and posterior leaflets [10]. The antero-superior leaflet retains a near-normal hinge-point position. It usually has multiple attachments to the RV wall and is typically elongated, showing a “sail-like” appearance on CCTA (Figure 1) [11,12]. The “atrialized” right ventricle refers to the thinned, stretched segment between the anatomic and functional TV annuli, which lacks normal contractile function (Figure 1). Poor leaflet coaptation results in TV regurgitation. As the disease advances, this typically leads to dilation of the right atrium, the atrioventricular junction, and the atrialized right ventricle. Elevated right atrial pressure frequently leads to right-to-left shunting through commonly associated defects such as PFO or ASD.

The clinical outcome in EA is largely determined by the degree of functional TV annular displacement [13]. This can be quantified by measuring the distance between the hinge points of the septal leaflet and the anterior leaflet of the mitral valve (Figure 1). A displacement index of >8 mm/m^2^ is a key diagnostic feature of EA and can be used to differentiate it from other types of TV dysplasia [13]. Surgical TV repair in EA is typically indicated for symptoms such as cyanosis, arrhythmias, heart failure, or progressive cardiomegaly [8,10].

### 4.2. TV Dysplasia (TVD)

**Figure 2:**
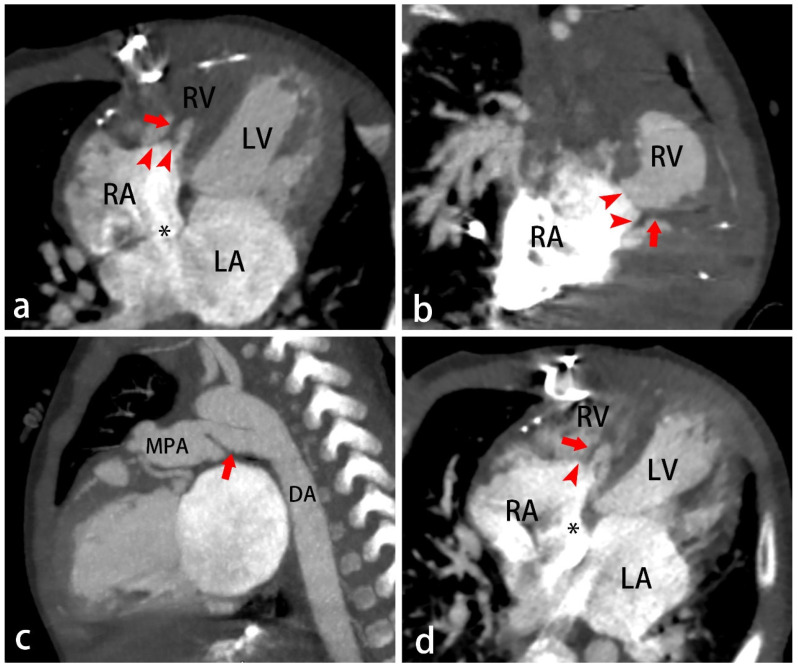
A 30-day-old boy with tricuspid valve dysplasia. CT angiography four-chamber view (**a**) and two-chamber view (**b**) show thickening of the tricuspid valve leaflets ((**a**,**b**), red arrowheads), which are connected to the thickened papillary muscle ((**a**,**b**), red arrows). The right ventricle is significantly hypoplastic and an atrial septal defect was also seen ((**a**,**d**), black asterisk). Maximum intensity projection (MIP) image shows the presence of patent ductus arteriosus (PDA) ((**c**), red arrow). CT angiography diastolic four-chamber view still shows restricted opening of the tricuspid valve leaflets (red arrowhead in (**d**)) and thickened papillary muscle ((**d**), red arrow). LV, left ventricle; LA, left atrium; RA, right atrium; RV, right ventricle; MPA, main pulmonary artery; DA, descending aorta.

Congenital TVD is a rare CHD that is frequently confused with EA. Although TVD lacks a precise definition, several morphological characteristics have been described in the literature. Unlike regurgitant EA, TVD has normal delamination of the leaflets, and the septal leaflet hinge point is in its normal location [14]. Consequently, TVD does not involve atrialization of the right ventricle. The three leaflets are almost invariably present. Additional characteristics include leaflet abnormalities, such as prolapse, thickening, rolling, or reduced height. The chordae tendineae can be shortened and thickened, restricting leaflet movement, or may be entirely absent, leading to an unsupported segment of a leaflet (Figure 2). The papillary muscles may also be hypoplastic or shortened [14]. Histopathologically, the thickened TV often correlates with fibrotic thickening of the fibrous layers, with some expansion of the spongy layer [14]. In TVD, the disorder of right ventricular myocardial development associated with EA is uncommon.

TV regurgitation is often well tolerated, and surgical intervention is indicated if symptoms of right heart failure or arrhythmias develop. While TVD lacks a standardized treatment approach, valve repair is the preferred surgical intervention after the neonatal period [15].

### 4.3. TA

TA accounts for approximately 1% of all CHDs [16]. It is characterized by total agenesis of the TV, and the right atrium is isolated from the hypoplastic right ventricle. In this condition, a PFO or ASD is required to allow deoxygenated blood to mix with oxygenated blood from the lungs, typically in the left atrium. To reach the lungs, blood may shunt through a VSD with univentricular heart physiology. If a VSD is absent or there is severe pulmonary obstruction, blood flow to the lungs depends on a PDA [4]. Most individuals (80%) with TA present with symptoms within the first month, and cyanosis is the most common clinical presentation. The severity of cyanosis is primarily determined by PBF, which depends on several factors: the degree of pulmonary obstruction, the presence and size of a VSD, the relationship of the great arteries, and whether a PDA is present [17].

**Figure 3:**
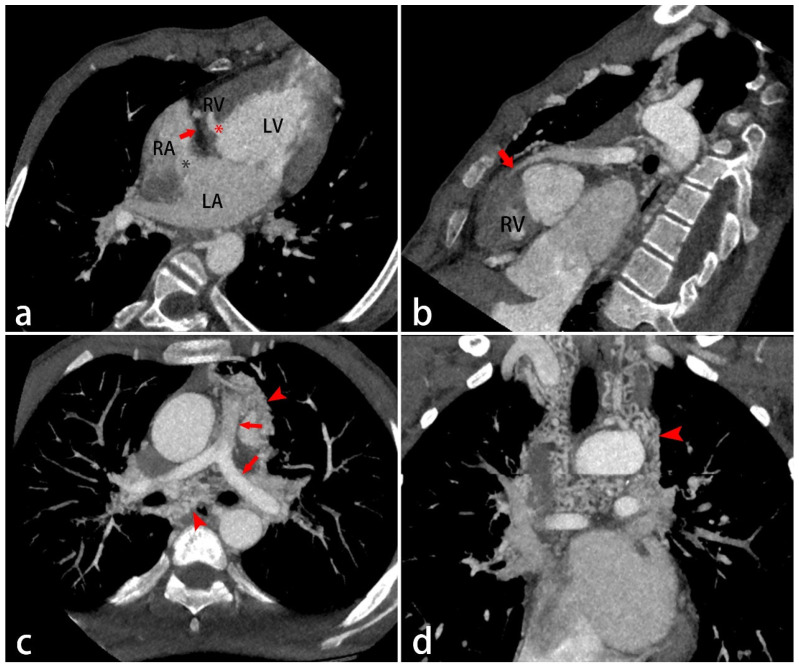
A 13-year-old boy with type I tricuspid atresia ((**a**), red arrow). Associated findings include ventricular septal defect ((**a**), red asterisk), atrial septal defect ((**a**), black asterisk), stenosis of the right ventricular outflow tract ((**b**), red arrow), and hypoplasia of the pulmonary arteries ((**c**), red arrows). Multiple systemic collaterals are present in the mediastinum and hila ((**c**,**d**), red arrowhead). LV, left ventricle; LA, left atrium; RV, right ventricle; RA, right atrium.

**Figure 4:**
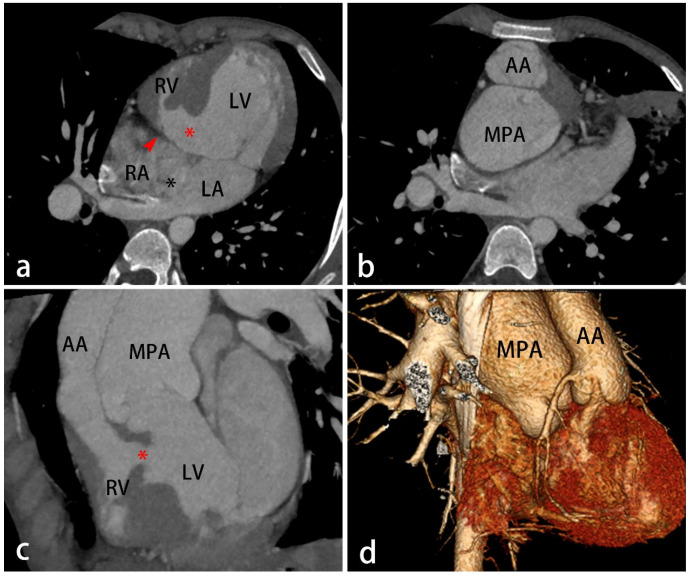
A 16-year-old male with type II tricuspid atresia ((**a**), red arrowhead). Associated findings include ventricular septal defect ((**a**,**c**), red asterisk) and atrial septal defect ((**a**), black asterisk). The ascending aorta lies anterior to the main pulmonary artery (**b**). The main pulmonary artery originates from the left ventricle, while the ascending aorta arises from the right ventricle (**c**,**d**), indicating complete transposition of the great arteries. LV, left ventricle; LA, left atrium; RV, right ventricle; RA, right atrium; AA, ascending aorta; MPA, main pulmonary artery.

TA is classified into four main types based on three key anatomical features: the relationship of the great arteries, the presence of a VSD, and the degree of pulmonary obstruction. Type I (69%) is characterized by normal anatomy of the great arteries (Figure 3). Type II (28%) involves D-transposition of the great arteries (D-TGA) (Figure 4). Type III (3%) involves great artery positions other than D-transposition. Type IV encompasses malposition defects, such as truncus arteriosus [17]. CT angiography can evaluate TV morphology and display the relationship between the heart and great vessels, making it valuable for clinical management.

The treatment principle is to establish a functional circulatory pathway through a series of staged palliative surgeries. The final surgical step is the Fontan procedure, which completes the circulation and allows for improved PBF [4].

## 5. Right Ventricular Outflow Obstructions

### 5.1. Isolated Congenital Pulmonary Valve Stenosis

**Figure 5:**
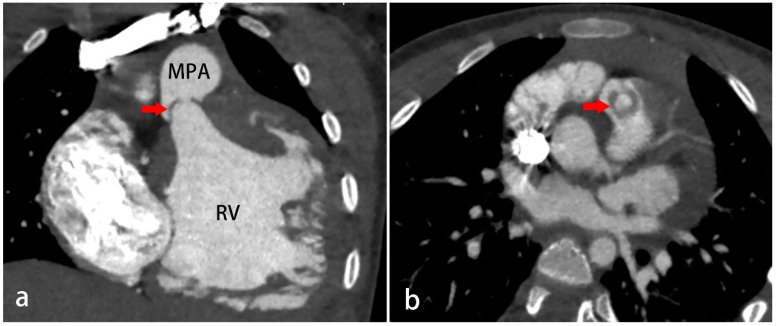
A 3-year-old girl with dome-shaped pulmonary valve. The pulmonary valve is upward bulging into the main pulmonary artery ((**a**), red arrow), resulting in a dome-shaped configuration with a small, round orifice ((**b**), red arrow) and right ventricular dilation. RV, right ventricle; MPA, main pulmonary artery.

**Figure 6:**
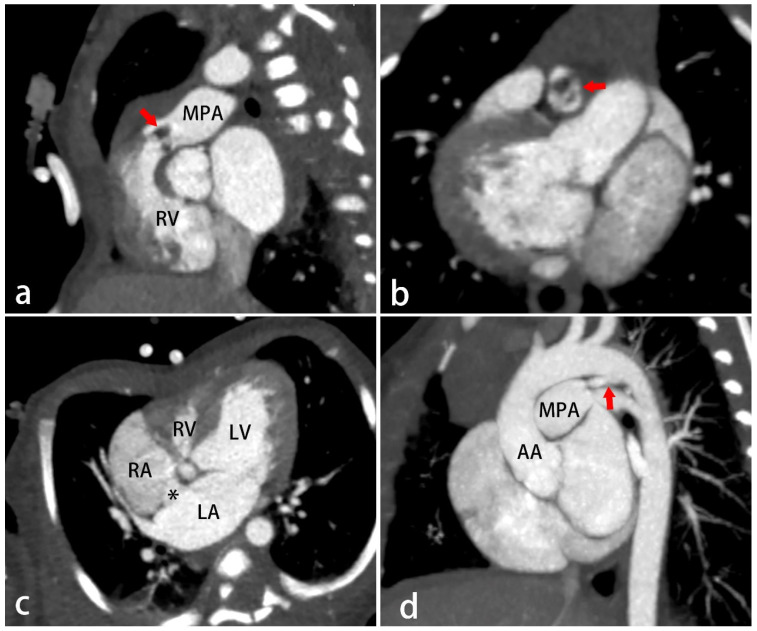
A 7-day-old boy with pulmonary valve dysplasia. CT angiography oblique images show a bicuspid pulmonary valve with irregular thickening of the leaflets and stenosis of the valve orifice ((**a**,**b**), red arrow). Four-chamber view demonstrates tricuspid atresia, hypoplastic right ventricle, and atrial septal defect ((**c**), black asterisk). Maximum intensity projection (MIP) image shows the presence of PDA ((**d**), red arrow). LV, left ventricle; LA, left atrium; RV, right ventricle; RA, right atrium; AA, ascending aorta; MPA, main pulmonary artery.

**Figure 7:**
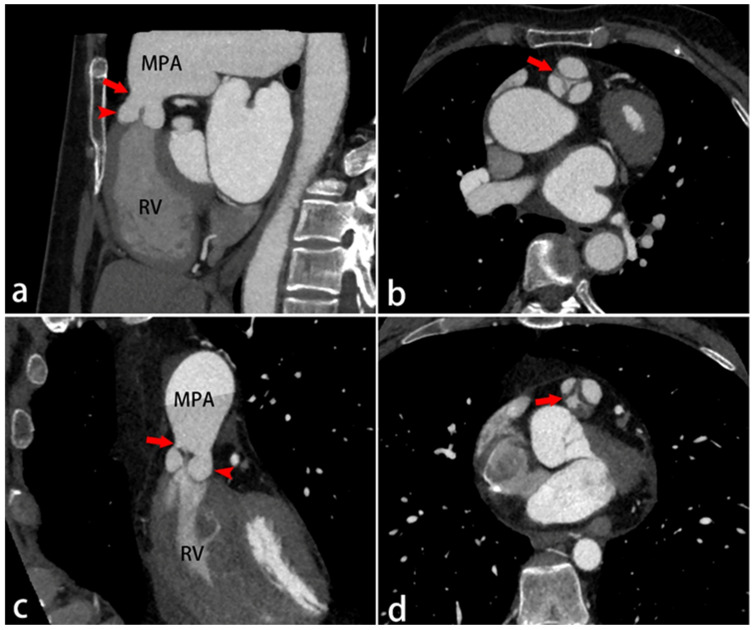
Hourglass-shaped stenosis of the pulmonary valve. (**a**,**b**) A 17-year-old female with localized thickened and narrowing of the pulmonary artery at the level of the valve orifice ((**a**,**b**) red arrow), creating an hourglass shape between the right ventricular outflow tract and post-stenotic dilation of the main pulmonary artery. The pulmonary sinuses are deep and bottle-shaped ((**a**), red arrowhead). (**c**,**d**) A 16-year-old male with thickened cusps ((**c**,**d**) red arrow), resulting in pulmonary valve stenosis and an hourglass-shaped configuration. The pulmonary sinuses show a bottle-shaped appearance ((**c**), red arrowhead). RV, right ventricle; MPA, main pulmonary artery.

Isolated congenital pulmonary valve stenosis, comprising 8–10% of CHDs, is the leading cause of RVOT obstruction [18,19]. It manifests in three main anatomical types. The first type is a dome-shaped pulmonary valve (40–60%). It is the result of commissural fusion with two to four raphes, creating upward bulging into the pulmonary artery. This results in a funnel-like structure with a small, round orifice (Figure 5). The size and shape of this opening depend on how thick and stiff the fused valve leaflets are [19]. The second type is valvular dysplasia (20–30%). It is characterized by thickened, immobile myxomatous cusps limited to the free edge of the leaflets that cause obstruction, despite minimal fusion of the commissures (Figure 6). This type of pulmonary stenosis is associated with a rather narrow valve annulus [19]. The third type is an hourglass deformity. This type shows localized thickening and narrowing of the pulmonary artery at the level of the valve orifice, creating an hourglass shape between the wider origin from the RVOT and the post-stenotic dilation of the pulmonary artery (Figure 7). This structural deformity, combined with thickened, rolled cusps, results in obstruction. In this type, the commissures show little to no fusion, and the valve sinuses are deep and bottle-shaped [20].

While dome-shaped pulmonary valves are effectively treated with balloon valvuloplasty (indicated for symptomatic patients with a peak gradient > 50 mmHg), dysplastic valves frequently require surgical replacement [21].

### 5.2. Tetralogy of Fallot (TOF)

**Figure 8:**
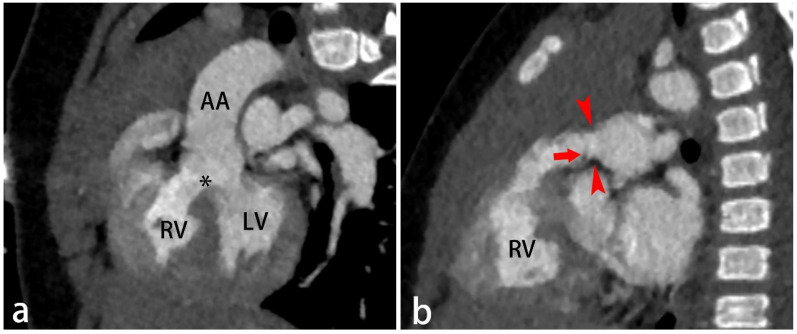
A 5-month-old boy with tetralogy of Fallot. CT angiography oblique image shows a large subaortic ventricular septal defect ((**a**), black asterisk), with overriding of the ascending aorta above the ventricular septal defect. The pulmonary valve shows a dome-shaped appearance ((**b**), red arrow), and supravalvular pulmonary stenosis is seen ((**b**), red arrowheads). The right ventricular wall is hypertrophied. AA, ascending aorta; RV, right ventricle; LV, left ventricle.

TOF is the most common cyanotic CHD, accounting for 4–8% of all congenital heart defects [22,23]. It is classically defined by four key features: (1) a large malalignment VSD, (2) RVOT obstruction or pulmonic stenosis, (3) an overriding aorta, and (4) right ventricular hypertrophy (Figure 8). The exact embryological cause of TOF remains unclear. It is appreciated that the formation of a perimembranous VSD leads to secondary displacement of the aorta, causing it to “override” the defect. This contributes to varying degrees of RVOT obstruction. The resulting increased resistance causes right-to-left intracardiac shunting. Consequently, the right ventricle undergoes remodeling, including hypertrophy, dilation, and fibrosis [24].

The obstruction of conventional TOF can be located at the subvalvular, valvular, or supravalvular level, including the main pulmonary artery and its branches, with severity ranging from mild to severe. Associated anomalies include a right aortic arch (25%), an ASD (Pentalogy of Fallot; 15%), a left superior vena cava (11%), and coronary abnormalities (5%) [25,26]. Specific variations of TOF encompass all types of pulmonary atresia with VSD (PAVSD) and absent pulmonary valve syndrome [22]. PAVSD is regarded as a severe subtype of TOF. Its key distinction from conventional TOF is the complete lack of an RVOT compared with the stenotic but patent pathway in TOF [22].

The severity of hypoxemia depends directly on PBF, which is determined by the degree of RVOT obstruction and alternative sources of flow such as a PDA or systemic-to-pulmonary collaterals. CCTA is valuable for evaluating the location and degree of pulmonary stenosis, aortic and great vessel origins, systemic-to-pulmonary collaterals, coronary anatomy, and other associated anomalies.

Complete surgical correction is the definitive treatment, typically performed between 3 and 6 months of age. It involves closing the ventricular septal defect and relieving right ventricular outflow tract obstruction. High-risk symptomatic TOF neonates require palliative care as a bridge to definitive surgery. Both transcatheter palliation and surgical options (systemic-to-pulmonary shunts, RVOT patches, right ventricle-to-pulmonary artery conduits) are applicable [23].

### 5.3. PAVSD

**Figure 9:**
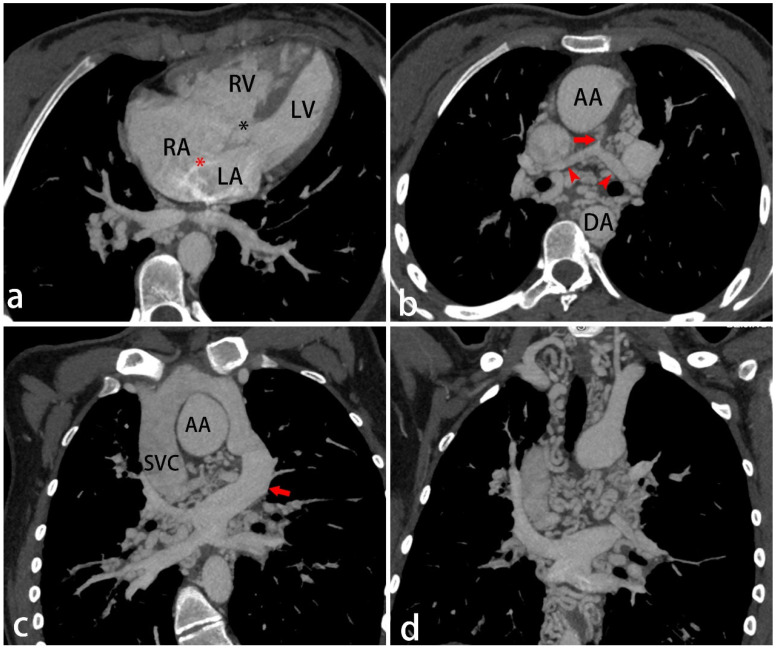
A 16-year-old female with pulmonary atresia with ventricular septal defect. Axial CT angiography image shows atresia of the main pulmonary artery ((**b**), red arrow) and hypoplastic left and right pulmonary arterial trunks ((**b**), red arrowheads), showing “seagull in flight” appearance. The four-chamber view shows a ventricular septal defect ((**a**), black asterisk) and an atrial septal defect ((**a**), red asterisk). There is anomalous pulmonary venous drainage into the superior vena cava ((**c**), red arrow). Multiple systemic collateral vessels are seen within the mediastinum (**d**). LV, left ventricle; LA, left atrium; RV, right ventricle; RA, right atrium; AA, ascending aorta; DA, descending aorta; SVC, superior vena cava.

PAVSD is a highly heterogeneous congenital heart defect characterized by discontinuity between the right ventricle and pulmonary arteries, along with a VSD. Patients may exhibit an undeveloped pulmonary valve or pulmonary artery. The pulmonary blood supply typically originates from extracardiac sources, most commonly via a PDA or major aortopulmonary collateral arteries (MAPCAs). The septal defects are typically large, subaortic, and of the membranous type [27]. PAVSD may involve various cardiac anomalies, including intracardiac defects such as TOF, double-outlet right ventricle, or transposition of the great arteries [28]. Associated aortic anomalies may include a right-sided aortic arch, aberrant subclavian artery, coarctation of the aorta, or double aortic arch [29]. Pulmonary venous anomalies, such as partial or total anomalous pulmonary venous drainage, may also occur [30] (Figure 9).

The pulmonary blood supply distribution in PAVSD depends on the presence of native pulmonary arteries (NPAs), a PDA, and MAPCAs. It determines the complexity of surgery, prognosis, and patient outcomes. PAVSD is classified into three types. In type A, there are only NPAs, which are supplied by the PDA. In type B, the PBF is supplied by both NPAs and MAPCAs. In type C, the PBF is supplied entirely by MAPCAs, with absent NPAs [31]. MAPCAs typically arise from the descending thoracic aorta, though origins from the subclavian, coronary, or abdominal aorta are possible. They usually anastomose with the intrapulmonary arteries near the hilum [28]. Other collateral sources, such as paramediastinal or intercostal arteries, are generally smaller and more numerous than MAPCAs [32] (Figure 9).

The confluent pulmonary arteries and shortened main pulmonary trunk resemble a “seagull in flight” on the frontal view of CCTA [28] (Figure 9). Key measurements include the length of the atresia segment, the presence of pulmonary artery confluence, and the diameters of the main, right, and left pulmonary arteries at their origin and the hilum, which are important for complete anatomical delineation and planning for treatment. Surgical management of PAVSD depends on age, cyanosis severity, RVOT obstruction, VSD size, pulmonary artery anatomy, and associated anomalies. Procedures include unifocalization, aortopulmonary shunts, RVOT patches, and the Glenn operation [32].

### 5.4. Pulmonary Atresia with Intact Ventricular Septum (PAIVS)

**Figure 10:**
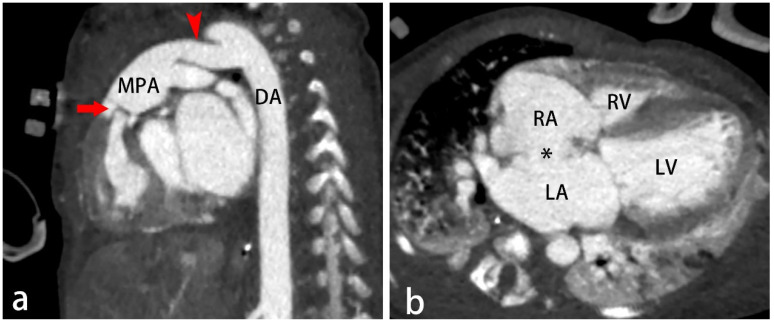
A 10-day-old girl with pulmonary atresia with intact ventricular septum. Oblique sagittal maximum intensity projection (MIP) CT angiography shows pulmonary valve atresia ((**a**), red arrow) and dilated main pulmonary artery, which is supplied by the aorta via a patent ductus arteriosus ((**a**), red arrowhead). The CT angiography four-chamber view shows an intact ventricular septum and an atrial septal defect ((**b**), black asterisk). LV, left ventricle; LA, left atrium; RV, right ventricle; RA, right atrium; DA, descending aorta; MPA, main pulmonary artery.

**Figure 11:**
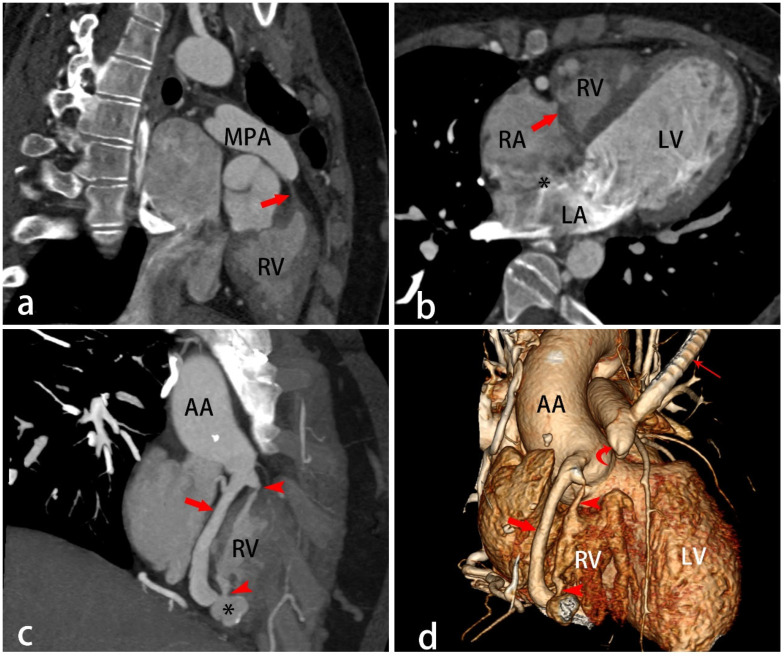
A 17-year-old female with pulmonary atresia with intact ventricular septum. CT angiography oblique sagittal view shows atresia of the right ventricular outflow tract ((**a**), red arrow). Four-chamber view reveals tricuspid atresia ((**b**), red arrow), atrial septal defect ((**b**), black asterisk), and intact ventricular septum. The coronary artery is dilated ((**c**,**d**), red arrow) with aneurysm formation ((**c**), black asterisk), and right ventricle–coronary artery fistulas are present ((**c**,**d**), red arrowheads). CT three-dimensional volume-rendered images demonstrate no connection between the right ventricle and the main pulmonary artery ((**d**), red curved arrow). A Blalock–Taussig (B–T) shunt was performed to improve pulmonary blood flow ((**d**), thin red arrow). LV, left ventricle; LA, left atrium; RV, right ventricle; RA, right atrium; AA, ascending aorta; MPA, main pulmonary artery.

PAIVS is a rare complex cyanotic CHD, comprising less than 1% of all CHDs [33]. It is characterized by membranous atresia of the pulmonary valve or muscular atresia of the RVOT with no interventricular communication, resulting in complete obstruction of right ventricular outflow [34] (Figure 10 and Figure 11). It is accompanied by varying degrees of hypoplasia of the TV and right ventricle and sometimes by a fistula connecting the right ventricle to the coronary circulation (Figure 11). The precise morphogenesis of PAIVS remains unclear. Some evidence suggests that pulmonary valve atresia may not be the primary event but rather a consequence of abnormal coronary arterial development [35].

The hemodynamics of patients with PAIVS are closely associated with abnormal cardiac morphology. Patients typically develop either tricuspid regurgitation or right ventricle–coronary fistulas, which help decompress the hypertensive right ventricle [36,37]. An ASD is required to allow right-to-left shunting of systemic venous return (Figure 9), leading to complete mixing on the left side of the heart and the expected cyanosis [36]. PBF depends entirely on the arterial duct; therefore, prenatal diagnosis enables the prompt initiation of prostaglandin E1 (PGE1) therapy to maintain ductal patency after birth. Coronary artery abnormalities include ventriculocoronary connections and coronary artery stenosis or atresia. Right ventricle-dependent coronary circulation (RVDCC) often occurs when coronary stenosis or occlusion occurs proximal to a fistulous connection [33]. In this subset, myocardial perfusion largely depends on right ventricular pressure, rendering patients vulnerable to ischemia or infarction, especially if the high-pressure right ventricle is decompressed [36,38].

CTA is an essential imaging modality for evaluating patients with PAIVS, offering unique value in assessing coronary abnormalities associated with RVDCC. It can also clearly display the morphology of the ASD, right ventricle, TV, and pulmonary arteries, which is helpful for determining interventional and surgical management. PAIVS management depends on anatomical features and RVDCC status. Prostaglandin E1 is used universally after birth. Patients free of RVDCC receive right ventricular decompression to pursue biventricular repair, with ductal stenting or surgical shunts as adjuncts. Those with RVDCC cannot undergo decompression and are treated with single-ventricle palliation or heart transplantation [33].

### 5.5. Proximal Interruption of the Pulmonary Artery (PIPA)

**Figure 12:**
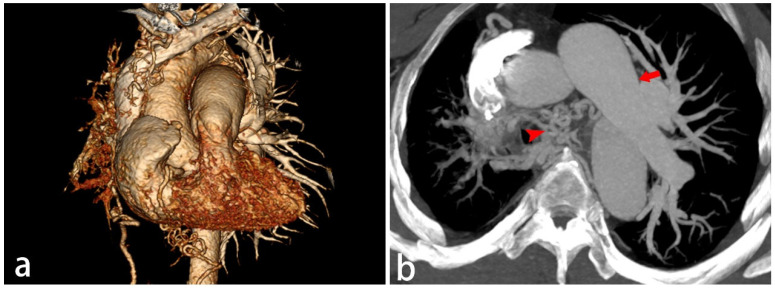
A 18-year-old male with proximal interruption of the right pulmonary artery. Volume-rendered CT angiography (**a**) and maximum intensity projection (MIP) image of CT angiography (**b**) show that the left pulmonary artery is in the normal position ((**b**), red arrow). The aortic arch is left-sided. Multiple dilated and tortuous systemic collateral vessels are seen in the mediastinum and right hilum ((**b**), arrowhead).

**Figure 13:**
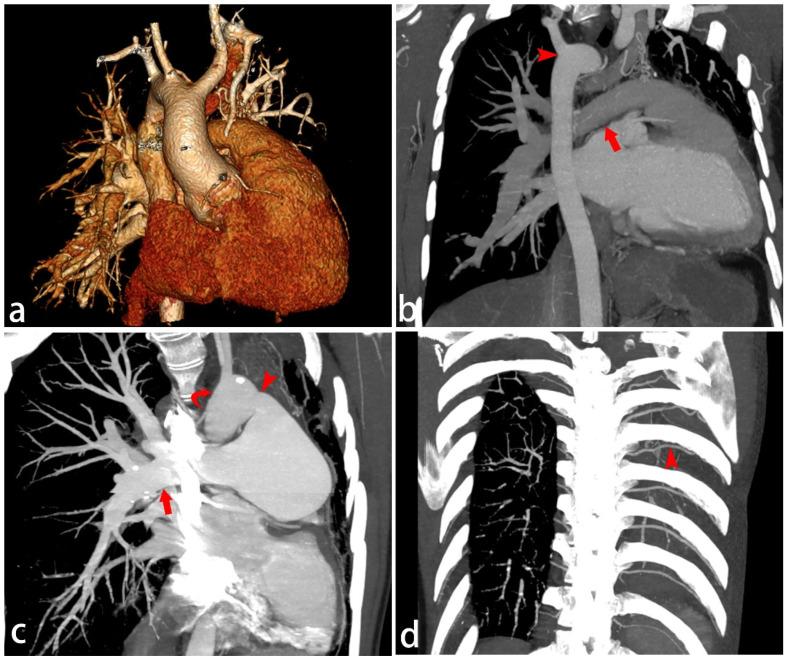
(**a**,**b**) A 15-year-old male with proximal interruption of the left pulmonary artery. Volume-rendered CT angiography (**a**) and maximum intensity projection (MIP) image of CT angiography (**b**) show the right-sided aortic arch ((**b**), red arrowhead). The right pulmonary artery is normal ((**b**), red arrow). (**c**,**d**) A 17-year-old female with proximal interruption of the left pulmonary artery. The aortic arch is left-sided ((**c**), red curved arrow). The right pulmonary artery is in the normal position ((**c**), red arrow) and there is an associated patent ductus arteriosus ((**c**), red arrowhead). The left intercostal arteries are dilated, indicating systemic collateral arteries formation ((**d**), red arrowhead).

PIPA is a rare developmental anomaly, occurring in approximately 1 out of every 200,000 young adults [39], but it can also be found in children. It is characterized by underdevelopment of the proximal segment of the pulmonary artery, while the intrapulmonary portions remain intact. It typically occurs in isolation and more frequently on the side opposite the aortic arch (Figure 12). Left pulmonary artery interruption is commonly associated with congenital anomalies such as TOF, a right aortic arch, septal defects, and PDA [40] (Figure 13). Although it abruptly terminates at the hilum, it is not truly absent. The intrapulmonary vessels remain relatively normal and are supplied by systemic collateral arteries. Embryologically, PIPA is thought to result from involution of the proximal sixth aortic arch (the normal origin of the proximal pulmonary artery), with persistence of the intrapulmonary artery connection to the distal sixth arch [19,40].

PIPA is usually an incidental, asymptomatic finding. Symptoms, when present, typically include progressive dyspnea, exercise intolerance, chest pain, or recurrent infections. Pulmonary hypertension or hemoptysis, which are potentially due to ruptured collateral vessels, may also occur [40,41]. CTA can not only provide anatomical detail of the involved vessels and surrounding thoracic structures but also assess lung parenchyma and collateral vascular formation. Asymptomatic or mildly symptomatic patients receive conservative management. For patients with obvious hemoptysis, transcatheter collateral vessel embolization is the first-line therapy. Pneumonectomy is reserved for refractory massive hemoptysis after failed embolization [40].

## 6. Summary

In conclusion, pediatric congenital right heart obstructive lesions are characterized by impaired blood flow through the right heart to the pulmonary artery. They encompass a spectrum of diseases including right ventricular inflow obstruction and right ventricular outflow obstruction (Table 1). Non-invasive CCTA is a valuable tool for the diagnosis and treatment planning of these lesions.

## Figures and Tables

**Table 1:** Characteristics, associated anomalies and treatment strategies of pediatric congenital right heart obstructive lesions.

Congenital Lesions	Characteristic Findings	Common Associated Anomalies	Surgical/Interventional Implications
Ebstein anomaly	Inferior displacement of the septal and posterior leaflets of TV; “Atrialized” right ventricle	PFO; ASD	Surgical TV repair for symptoms such as cyanosis, arrhythmias, heart failure, or progressive cardiomegaly
TV dysplasia	TV abnormalities (prolapse, thickening, rolling, or reduced height); Chordae tendineae and papillary muscle abnormalities	ASD; PDA	Surgical intervention for symptoms of right heart failure or arrhythmias
Tricuspid atresia	Total agenesis of the TV; No connection between right atrium and hypoplastic right ventricle	PFO or ASD; VSD; PDA; TGA	Final surgical step is the Fontan procedure to improved PBF
Isolated congenital pulmonary valve stenosis	Dome-shaped pulmonary valve; Pulmonary valvular dysplasia; Hourglass deformity	TOF	Balloon valvuloplasty for dome-shaped pulmonary valves; Surgical replacement for pulmonary valvular dysplasia
Tetralogy of Fallot	A large malalignment VSD; RVOT obstruction or pulmonic stenosis; An overriding aorta; Right ventricular hypertrophy	ASD; Coronary abnormalities	Complete surgical correction at 3 to 6 months of age; High-risk symptomatic neonates require palliative care
Pulmonary atresia with VSD	Discontinuity between right ventricle and pulmonary arteries; VSD	TOF; PDA; Double-outlet right ventricle; TGA; Aortic anomalies; Pulmonary venous anomalies	Surgical management depends on age, cyanosis severity, RVOT obstruction, VSD size, pulmonary artery anatomy, and associated anomalies
Pulmonary atresia with intact ventricular septum	Complete obstruction of right ventricular outflow; Intact ventricular septum	TV and right ventricle Hypoplasia; RVDCC; ASD; PDA	Patients free of RVDCC receive right ventricular decompression, ductal stenting or surgical shunts; Single-ventricle palliation or heart transplantation for RVDCC
Proximal interruption of the pulmonary artery	Underdevelopment of the proximal segment of the pulmonary artery; Intrapulmonary vessels relatively normal	TOF; VSD; PDA	Conservative care is used for mild PIPA; Transcatheter embolization treats hemoptysis, while pneumonectomy is for refractory bleeding

TV, tricuspid valve; PFO, patent foramen ovale; ASD, atrial septal defect; VSD, ventricular septal defect; PDA, patent ductus arteriosus; TGA, transposition of the great arteries; RVOT, right ventricular outflow tract; TOF, tetralogy of Fallot; RVDCC, right ventricle-dependent coronary circulation; PIPA, proximal interruption of the pulmonary artery.

## Data Availability

No new data were created or analyzed in this study. Data sharing is not applicable to this article.
